# Host–pathogen coevolution in human tuberculosis

**DOI:** 10.1098/rstb.2011.0316

**Published:** 2012-03-19

**Authors:** Sebastien Gagneux

**Affiliations:** Department of Medical Parasitology and Infection Biology, Swiss Tropical and Public Health Institute and University of Basel, Socinstrasse 57, 4002 Basel, Switzerland

**Keywords:** mycobacteria, infection, virulence, genotyping, phylogeography, fitness

## Abstract

Tuberculosis (TB) is a disease of antiquity. Yet TB today still causes more adult deaths than any other single infectious disease. Recent studies show that contrary to the common view postulating an animal origin for TB, *Mycobacterium tuberculosis* complex (MTBC), the causative agent of TB, emerged as a human pathogen in Africa and colonized the world accompanying the Out-of-Africa migrations of modern humans. More recently, evolutionarily ‘modern’ lineages of MTBC expanded as a consequence of the global human population increase, and spread throughout the world following waves of exploration, trade and conquest. While epidemiological data suggest that the different phylogenetic lineages of MTBC might have adapted to different human populations, overall, the phylogenetically ‘modern’ MTBC lineages are more successful in terms of their geographical spread compared with the ‘ancient’ lineages. Interestingly, the global success of ‘modern’ MTBC correlates with a hypo-inflammatory phenotype in macrophages, possibly reflecting higher virulence, and a shorter latency in humans. Finally, various human genetic variants have been associated with different MTBC lineages, suggesting an interaction between human genetic diversity and MTBC variation. In summary, the biology and the epidemiology of human TB have been shaped by the long-standing association between MTBC and its human host.

## 1. Introduction

Tuberculosis (TB) remains a major global health problem. Ten million new TB cases and 2 million deaths are estimated to occur each year, more than any time in history [1]. Furthermore, an estimated 2 billion people are thought to be latently infected, providing a large reservoir for active TB that will last for decades [2]. Even though during the last centuries TB used to cause up to half of all human deaths in Europe and North-America [3], today the disease affects primarily the developing world. Notably, some of the largest emerging economies including China and India remain among the 22 high-burden countries for TB identified by the World Health Organization (WHO). These two countries collectively accounted for more than one-third of all new TB cases in 2009 [1].

TB is the prototype of a disease of poverty [4]. In addition to the well-established environmental and socio-economical risk factors for TB [5], recent times have witnessed the appearance of new forces driving the global TB pandemic: HIV/AIDS and the emergence of antimicrobial resistance. Since its initial appearance in the 1980s, the HIV/AIDS pandemic had a dramatic effect on TB incidence, particularly in sub-Saharan Africa [6]. Moreover, the introduction of chemotherapy against TB, which started in the 1940s, has led to increasing levels of drug resistance [7]. The global emergence of multi-drug resistance has initiated the post-antibiotic era, making TB essentially incurable in many parts of the world [8]. Left untreated TB kills 50 per cent of patients on average [9], with HIV co-infected patients facing an even higher risk of death [10]. The only available vaccine against TB is ‘bacille Calmette Guérin’ (BCG), an attenuated form of *Mycobacterium bovis*, which is a pathogen of cattle (further discussed below) [11]. However, BCG only protects young children against TB meningitis, the most severe form of the disease. Why BCG does not protect adults reliably against the classical pulmonary form of TB is unknown [12].

TB is an ancient disease [13]. Recent data show that, contrary to the traditional belief linking the origin of TB to the development of agriculture and animal domestication during the Neolithic transition approximately 10 000 years ago, TB has been affecting humans for much longer. Hence, the long-term interaction between the TB microbe and its human host raises the question as to whether coevolution has occurred and if it can be detected. Here, I start by summarizing some of the most important features of TB and the agents causing it. I then discuss several recent findings which support the notion of coevolution in human TB. Some of the points discussed are purely speculative. Yet, I am addressing them in the hope of fuelling future research.

## 2. Tuberculosis and the *Mycobacterium tuberculosis* complex

TB is caused by a group of phylogenetically closely related bacteria, collectively known as the *Mycobacterium tuberculosis* complex (MTBC) [14]. TB in humans is primarily caused by *M. tuberculosis* and *Mycobacterium africanum*, a phylogenetic variant of MTBC limited to West Africa [15]. In addition, several animal-adapted members of MTBC exist, which affect a range of wild and domestic animal species [16]. These include *M. bovis* (a pathogen of cattle), *Mycobacterium caprae* (sheep and goats), *Mycobacterium microti* (voles) and *Mycobacterium pinnipedii* (seals and sea lions). *Mycobacterium bovis* used to be a significant cause of human TB, primarily in children who consumed raw milk [17]. However, *M. bovis* infections in humans decreased markedly following the introduction of pasteurization and meat-control practices. Moreover, *M. bovis* does not easily transmit between humans and, similarly, while *M. tuberculosis* has been isolated from various animal species, including cattle, there is currently no evidence of animal-to-animal transmission of *M. tuberculosis* or *M. africanum*. Hence, the different members of MTBC appear to be best adapted to their particular host species [18].

**Figure 1.**
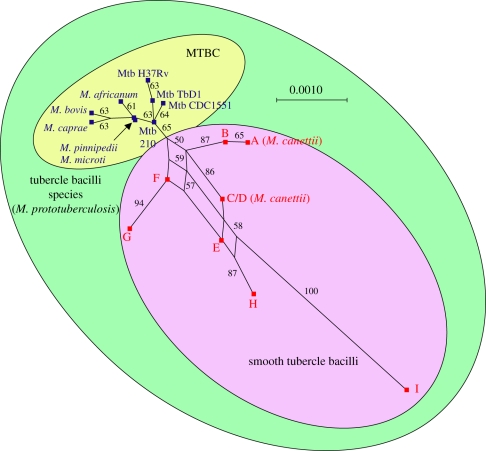
Phylogenetic relationship between ‘smooth’ tubercle bacilli and classical MTBC. Multi-locus sequence data of six housekeeping genes show that the smooth tubercle bacilli are more genetically diverse than MTBC. The network structure of the phylogenetic relationships between members of the smooth tubercle bacilli indicates ongoing horizontal gene exchange, which is absent in MTBC. Adapted from Gutierrez *et al*. [19].

One particular member of MTBC deserves special mention: *Mycobacterium canettii*, which is part of the so-called ‘smooth TB bacilli’ [19]. This somewhat enigmatic microbe was first described in the 1960s, and so far only about 60 isolates have been reported [20]. Intriguingly, the large majority of these were recovered from TB patients in Djibouti or from individuals who spent some time at the Horn of Africa prior to developing TB. *Mycobacterium canettii* differs in many ways from classical MTBC. This organism produces smooth and shiny colonies on solid growth media, which are distinct from the rough colony morphology characteristic of classical MTBC. Moreover, *M. canettii* and the other ‘smooth TB bacilli’ harbour much more genetic diversity compared with classical MTBC (figure 1). Most importantly, *M. canettii* shows clear evidence of ongoing horizontal gene exchange (figure 1), which does not occur in classical MTBC [21,22]. This latter observation, combined with the lack of evidence for human-to-human transmission of *M. canettii*, and the fact that *M. cannettii* clinical isolates are rare, suggests that this organism is an opportunist, and that an environmental reservoir exists somewhere in the Horn of Africa [23].

By contrast, *M. tuberculosis* and *M. africanum* are obligate human pathogens with limited survival outside of the human body and no known animal reservoir [15]. Moreover, these microbes have to cause active disease in order to transmit to secondary hosts [24]. In other words, in MTBC there is a direct link between virulence and transmission, which is in contrast to many other pathogens [25,26]. Because latently infected individuals are considered non-infectious [2], global efforts in controlling TB almost exclusively focus on detecting and treating patients with active TB [1]. However, because culture facilities are often not available, most places still diagnose TB by sputum-smear microscopy. Unfortunately, this technique suffers from limited sensitivity and up to 50 per cent of active TB cases are routinely missed. Individuals harbouring the largest numbers of acid-fast bacilli in their sputum are considered the most infectious [27].

MTBC can infect virtually any organ. However, from a public health point of view, classical pulmonary TB is the most important form of the disease because of its infectious nature. One of the main immunopathological features of active pulmonary TB is the formation of lung cavities. These structures tend to harbour large numbers of bacteria, which is why cavitary TB tends to be the most infectious form of the disease. By contrast, extrapulmonary TB is generally non-infectious and therefore has a lower public health priority [1].

## 3. The origin of human tuberculosis

Until a few years ago, the prevalent view was that TB originated in animals and was transferred to humans during the Neolithic transition [28]. However, comparative genomics and population genetic studies have challenged this notion [29,30]. In particular, *M. bovis* and the other animal-adapted members of MTBC have a genome which is about 60 000 base pairs smaller than the genome of the human-adapted *M. tuberculosis* [14,31]. Because MTBC exhibits essentially no ongoing horizontal gene exchange [21], the genomic regions present in *M. tuberculosis* but absent from *M. bovis* represent deletions in the latter rather than insertions in the former. More recently, phylogenetic analyses of 89 gene concatenates in 108 strains representative of the global MTBC diversity revealed that the animal-adapted members of the MTBC clustered together, representing an ingroup with respect to the global phylogeny of MTBC, while all other taxa represented human-adapted forms [32]. In summary, although *M. tuberculosis* and *M. bovis* do share a common ancestor, the most parsimonious scenario suggests that humans gave TB to animals rather than the other way around [28]. This latter observation also suggests that TB in humans pre-dates the Neolithic transition.

That human TB appears to be significantly older than approximately 10 000 years is supported by several additional lines of evidence. A recent study from Turkey reported a 500 000 year old fossil of *Homo erectus*, which shows lesions characteristic of TB [33]. Maybe not surprisingly, this finding is controversial, as it suggests that TB predated the origin of modern humans. Similarly, a study by Gutierrez *et al*. [19] proposed that *M. canettii* and the other smooth TB bacilli represent a group of organisms likely to contain the ancestor of MTBC. The authors used DNA sequence data of six housekeeping genes and calculated an approximate age of 3 million years for the origin of what they have been referring to as ‘*Mycobacterium prototuberculosis’* (figure 1). However, these dating results, too, have been contested [34].

**Figure 2.**
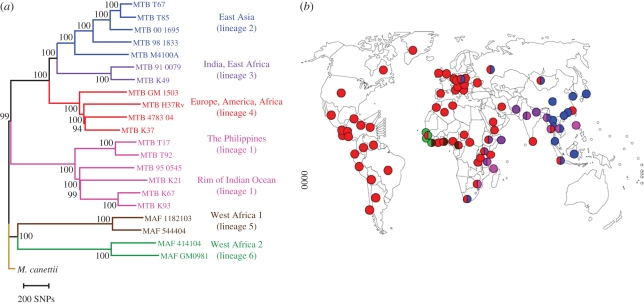
Global phylogeography of MTBC. (*a*) Global phylogeny of human-adapted MTBC is based on 22 whole-genome sequences. Adapted from Comas *et al*. [35]. (*b*) Global distribution of the six main human-adapted MTBC lineages. Coloured dots represent the dominant MTBC lineage(s) in each country. Adapted from Gagneux *et al*. [36].

More recently, Comas *et al*. [35] generated the first whole-genome-based global phylogeny of human-adapted MTBC (figure 2*a*). This analysis revealed that the two MTBC lineages generally referred to as *M. africanum* (lineage 5 and 6 in figure 2*a*) are the most basal. These two lineages occur almost exclusively in West Africa for reasons unknown (figure 2*b*) [15]. Furthermore, Africa is the only region of the world that harbours all six main human-adapted MTBC lineages (figure 2*b*) [37]. In other words, Africa harbours the largest diversity of human-adapted MTBC in the world. Both of these observations are reminiscent of the situation in modern humans; i.e. *Homo sapiens* from Africa are known to be phylogenetically ‘ancestral’ and harbour most of the known human genetic diversity [38]. Finally, human-adapted MTBC exhibits a phylogeographic population structure with different lineages associated with different human populations (figure 2) [36].

**Figure 3.**
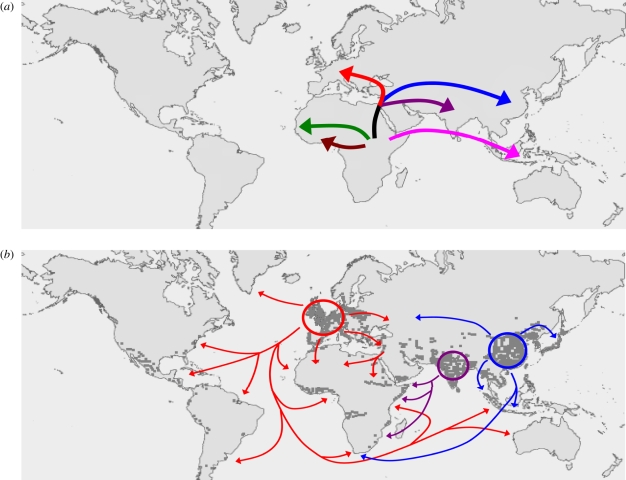
‘Out-of-and-back-to-Africa’ scenario for the evolutionary history of human TB. Adapted from Hershberg *et al*. [32]. (*a*) MTBC originated in Africa and some lineages accompanied the Out-of-Africa migrations of modern humans. (*b*) The three evolutionary ‘modern’ MTBC lineages seeded Europe, India and China, respectively, and expanded as a consequence of the sharp increases in human populations in these regions starting a few centuries ago (each dark grey dot corresponds to 1 million people). These lineages then spread throughout the world via exploration, trade and conquest. Lineage colours correspond to figure 2.

Based on these observations, Hershberg *et al*. [32] proposed an evolutionary scenario for the origin and global spread of human TB. This scenario was supported by multi-locus sequence data from 108 global MTBC strains and postulates that human MTBC originated in Africa and accompanied the Out-of-Africa migrations of modern humans approximately 70 000 years ago (figure 3). While two phylogenetically ‘ancient’ MTBC lineages remained in Africa, other lineages left Africa and spread into Eurasia where the three phylogenetically ‘modern’ lineages seeded Europe, India and China, respectively. These areas experienced strong human population growth during the last few centuries. As a consequence, the MTBC populations in these areas expanded, and concomitantly spread globally through waves of human exploration, trade and conquest. In support of this scenario, a study by Wirth *et al*. [39] based on mini-satellite data in another gobal collection of MTBC isolates detected molecular signatures of recent (i.e. less than 200 years) population expansion in the different MTBC populations. These signatures were more pronounced in MTBC populations from Europe and Asia compared with those from Africa.

Assuming the ‘Out-of-and-back-to-Africa’ scenario of TB evolution by Hershberg *et al* is correct, then several important questions remain. For a long time, the general view was that TB did not exist in the Americas before European contact [40]. This notion was to a large extent based on the high susceptibility to TB observed in Native Americans. This gave rise to the so-called ‘Virgin soil’ hypothesis, according to which Native Americans were immunologically naive because they (presumably) had not been exposed to TB before European contact [41]. This view has now changed quite radically, with an emerging consensus among historians and paleopathologists suggesting that TB was in fact present in pre-Columbian America [42]. Note that Native Americans remain highly susceptible to TB, but other, more general risk factors for TB are increasingly being invoked to explain this phenomenon, including poor living and nutritional conditions and alcohol abuse [41]. Even though ancient DNA studies were able to detect mycobacterial DNA in pre-Columbian human remains [43], the more detailed characteristics of this DNA remains unknown. In other words, we do not know what the exact a etiologic agent of TB was at the time [42]. Today, by far, most TB in the Americas is caused by lineage 4, also known as the Euro-American lineage (figure 2*b*) [37]. Future studies will tell whether pre-Columbian TB was caused by MTBC related to Asian forms, as would be expected given the original human colonization of the Americas via the Bering Strait. Alternatively, pre-Columbian TB might have been caused by mycobacterial lineages which are now extinct, perhaps because they were outcompeted by lineage 4/Euro-American lineage following the massive influx of Europeans with active or latent TB into the Americas between the early eighteenth and early twentieth century [44].

Another open question is why despite centuries of cross-Atlantic slave trade, *M. africanum* lineages are almost never isolated in the Americas [15]. It is reasonable to assume that a proportion of the 10–15 million African slaves taken to the Americas would have been latently infected with TB. Similar to the argument above, perhaps, *M. africanum* in the Americas was outcompeted by lineage 4 following the European exodus into America. Interestingly, there is evidence that at least in some areas of West Africa, *M. africanum* has been decreasing and *M. tuberculosis* increasing over the last few decades [45]. We will return to the phenotypic differences between *M. africanum* and ‘modern’ MTBC which could account for this phenomenon further below.

In summary, the available evidence suggests that human MTBC originated in Africa and has been infecting humans for millennia. Considering this long-standing host–pathogen association, one wonders whether some degree of coevolution might have occurred between MTBC and its human host.

## 4. Signs of host–pathogen coevolution in human tuberculosis

Host–pathogen coevolution can broadly be defined as ‘reciprocal, adaptive genetic changes in interacting host and pathogen species’ [46]. While such changes will be difficult to detect in humans, approaching the subject from the pathogen side appears more promising. Over recent years, data have accumulated suggesting that the different MTBC lineages might be adapted to different human populations. Not only does MTBC exhibit a global biogeographic population structure (figure 2*b*), but the associations between the particular MTBC lineages and human populations are maintained in cosmopolitan settings where human populations and their associated MTBC strains experience at least some degree of intermingling [21,47,48]. Moreover, a study in San Francisco found that MTBC lineages primarily transmitted among their sympatric host populations (figure 3) [36]. Intriguingly, transmission of allopatric strains was strongly associated with known risk factors for TB such as HIV co-infection. Whether these findings reflect true biological phenomena rather than mere sociological factors need to be explored further. Nevertheless, they are consistent with host-specific adaptation of MTBC lineages, which if confirmed, would also be consistent with past host–pathogen coevolution.

## 5. Immune subversion instead of evolutionary arms-race

**Figure 4.**
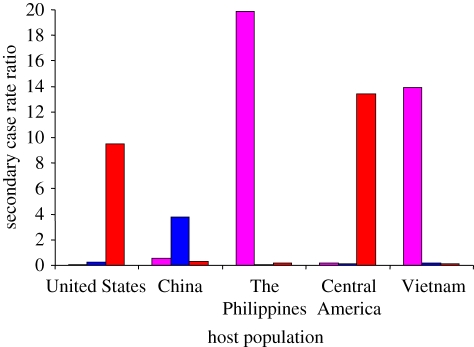
Preferential sympatric transmission of MTBC lineages in San Francisco. The number of secondary TB cases generated between 2001 and 2009 is plotted as a function of the number of index cases (secondary case rate ratio) and the human population in which these secondary cases occurred. In each human population, the number of secondary cases caused by sympatric MTBC lineages was significantly higher compared with the secondary cases caused by allopatric lineages. Sympatric and allopatric lineages were defined based on their usual phylogeographic association shown in figure 2. (Red, lineage 4 or Euro-American lineage; Blue, lineage 2 or East-Asian lineage; Pink, lineage 1 or Indo-Oceanc lineage.) Adapted from Gagneux *et al*. [36].

If host-specific pathogen adaptation has occurred in human MTBC, we would be most likely to detect the associated molecular changes in parts of the MTBC genome that interact with the human immune system, i.e. in genes encoding antigens. More generally, if host–pathogen coevolution is occurring in MTBC, we should be able to detect its signature in antigenic genes. One of the key features of host–pathogen coevolution in other disease systems is the ongoing evolutionary arms-race between the pathogen and the host immune system [49]. Many pathogenic viruses, bacteria and protozoa evade host immunity by varying their antigenic genes. Is MTBC using a similar strategy to evade human immune responses? Comas *et al*. [35] aimed at answering this question by comparing the genetic diversity in different gene classes of MTBC. The authors sequenced the genomes of 21 human MTBC strains representative of the global diversity of MTBC. As a proof of concept, Comas *et al*. first showed that, as expected, essential genes were more evolutionarily conserved than non-essential genes (figure 4). Surprisingly however, the authors found that the known human T-cell epitopes of MTBC were the most conserved regions of the MTBC genome (figure 4). In fact, more than 95 per cent of the 491 individual epitopes analysed had no amino acid change at all. Based on their findings, the authors speculated that the immune responses elicited by these T-cell epitopes might in fact be beneficial to the bacteria rather than to the host. In other words, rather than escaping host immunity, MTBC wants to be recognized because the ensuing host immune responses contribute to tissue destruction and the formation of cavities in the host lung, which ultimately enhances transmission [50]. The notion that T-cell immunity contributes to the formation of lung cavities in TB patients is supported by the observation that HIV-positive TB patients with low CD4 T-cell counts are less likely to present with cavitary TB than HIV-positive patients with higher CD4 cell counts [6]. In summary, the reason these T-cell epitopes are evolutionarily hyper-conserved might be that they perform an essential function in the sense of contributing to the successful spread of MTBC. This of course does not exclude the possibility that MTBC might harbour antigenic variation in other regions of its genome [51].

## 6. Human demography and the evolution of virulence

Despite the lack of genetic diversity in the known T-cell epitopes, much genetic diversity exists within and between MTBC lineages, which so far has not been explored experimentally [32]. This diversity could be at the basis of the phenotypic variation, which is increasingly being reported in human-adapted MTBC [52,53]. Our unpublished data from whole genome comparisons in MTBC show that two human-adapted MTBC strains can differ by more than 1700 single-nucleotide polymorphisms (SNPs), which is close to the genetic distance between *M. tuberculosis* and the cattle-adapted *M. bovis*. Furthermore, about two-thirds of these SNPs are non-synonymous (figure 4), and the majority of these non-synonymous SNPs are predicted to have a functional consequence [32]. Thus, any of these differences on their own, or in combination, could influence the various MTBC phenotypes [52].

As mentioned above, the six main human-adapted lineages of MTBC can be divided into evolutionarily ‘ancient’ and ‘modern’. As can be seen from figure 2, the three ‘modern’ lineages are overall more successful than the other lineages in terms of their geographical spread [37]. What could account for this differential success? According to the evolutionary scenario by Hershberg *et al*. [32] (figure 3), the ‘modern’ lineages expanded as a consequence of the sharp increases in human populations during the last few centuries in Europe, India and China. Ecological theory predicts that when virulence is positively correlated with transmission, as is the case in TB, access to a larger number of susceptible hosts favours higher virulence and a shorter latency period [26]. If we believe the proposed coevolutionary history of MTBC with humans, most of this evolutionary history would have occurred during hunter–gatherer times, when human population densities were low. In fact, it has been argued that the characteristic latency period of human TB, followed by reactivation and active disease several decades later, might have evolved to allow MTBC to access new birth cohorts of susceptible hosts and, at the same time, avoid a burn-out situation whereby all susceptible hosts would be decimated by an overly virulent pathogen [54]. By contrast, high host densities, similar to the ones encountered in the crowded cities of eighteenth and nineteenth century Europe [3], might have selected for less ‘prudent’ forms of MTBC [55], as access to susceptible hosts was no more a limiting factor. Based on this scenario, and because the ‘modern’ MTBC lineages were exposed to this rapid human expansion, we would predict that ‘modern’ MTBC will be more virulent and associated with a shorter latency period compared with ‘ancient’ MTBC. Intriguingly, several recent data from the laboratory and the field support this prediction.

## 7. From *Mycobacterium tuberculosis* complex genotype to phenotype

**Figure 5.**
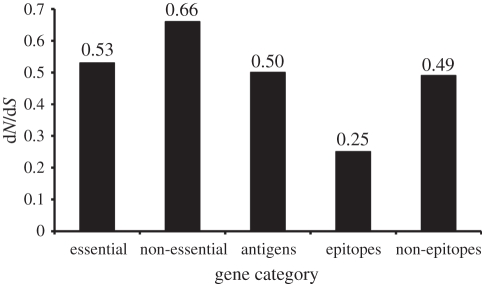
Human T-cell epitopes of MTBC are evolutionarily hyper-conserved. Ratio of non-synonymous mutations to synonymous mutations in different gene classes based on whole genome sequencing of 22 MTBC strains representative of the global MTBC diversity. Adapted from Comas *et al*. [35].

The first human cells encountered by MTBC during the course of an initial infection are believed to be alveolar macrophages [24]. These cells engulf MTBC but cannot destroy them. In fact, MTBC evolved mechanisms to inhibit endosome maturation and phagosome–lysosome fusion, which allows the bacteria to survive and even multiply within these macrophages [56]. Human monocyte-derived macrophages (MDMs) have thus been used as a model to study the cell biology and early mechanisms of infection in TB. Portevin *et al*. [57] recently used such a model to study the innate immune responses to 28 MTBC strains spanning the whole global diversity of human MTBC. The authors used MDMs from eight different healthy donors, infected them with one of each of these 28 strains, and measured a series of cytokines and chemokines after 24 h of infection. The results showed that the MDMs infected with different MTBC strains differed markedly in the levels of cytokines and chemokines produced. Interestingly, MDMs infected with ‘modern’ strains produced significantly less pro-inflammatory cytokines compared with ‘ancient strains’ (figure 5). The same results were obtained when infecting human monocyte-derived dendritic cells or bone marrow-derived murine macrophages. Furthermore, the results were reproducible across the eight human donors. Previous work has shown that low pro-inflammatory innate immune responses to MTBC infection were associated with a higher virulence in animal models [58,59]. Moreover, work from the 1960s showed that MTBC strains from South India were less virulent in guinea pigs than strains from the UK [60]. We now know that these South-Indian strains belonged to the ‘ancient’ lineage 1 while most strains in the UK belong to the ‘modern’ lineage 4 (figure 2).

**Figure 6.**
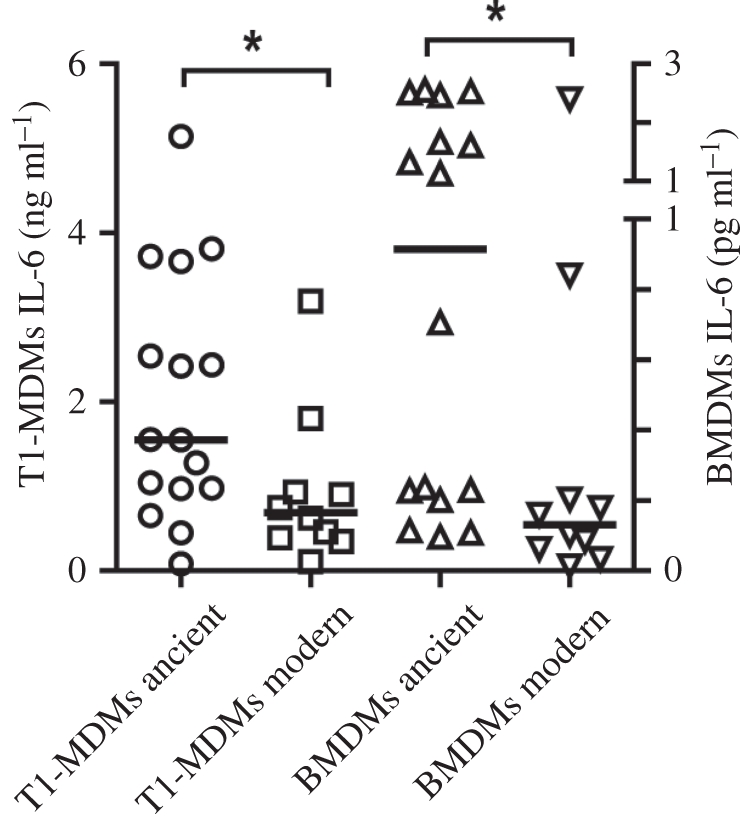
Evolutionarily ‘modern’ MTBC lineages are hypo-inflammatory in human and murine macrophages. Human monocyte-derived (MDM) and murine bone marrow-derived (BMDM) macrophages were infected with one of 28 MTBC strains representative of the global MTBC diversity. Cytokines were measured in supernatants after 24 h of infection. *p < 0.05, Mann–Whitney U test. Adapted from Portevin *et al*. [57].

In a molecular epidemiological study of TB transmission in the Gambia, de Jong *et al*. [61] recruited 317 TB patients and followed their 1808 HIV-negative household contacts during 2 years. The authors then categorized these contacts depending on the MTBC lineage the index case was infected with, and determined how many contacts would become newly infected (i.e. indicating transmission from the index case) and how many would develop active TB. The results showed that, while there was no difference in transmission, contacts exposed to ‘modern’ MTBC were more likely to develop active TB compared with individuals exposed to ‘ancient’ MTBC (figure 6).

Taken together, these observations are consistent with the notion that ‘modern’ strains have evolved a strategy of higher virulence and shorter latency, perhaps to better adapt to the increasing availability of susceptible hosts brought by the large human population increases of the last few centuries.

## 8. The role of human genetic diversity

**Figure 7.**
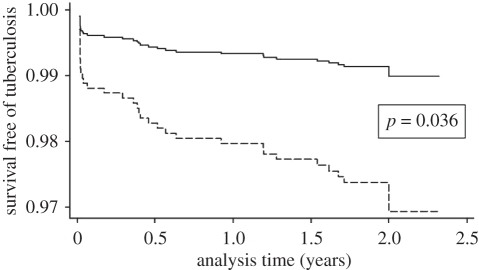
Evolutionarily ‘modern’ MTBC lineages are associated with a shorter latency in human TB. TB patients of 1808 HIV-negative household contacts were followed over 2 years. The number of active TB cases occurring in these contacts during the study period was determined and stratified by the MTBC lineage of the index cases (solid line, ‘ancient’ MTBC (i.e. *M. africanum*), dashed line, ‘modern’ MTBC. Adapted from de Jong *et al*. [61].

So far our discussion has primarily focused on the pathogen, maybe because the case for unilateral ‘host-specific pathogen adaptation’ might be more plausible and easier to demonstrate than true reciprocal ‘coevolution’ between MTBC and humans, given the shorter generation-time of the former. Nevertheless, variation in TB susceptibility across humans is well established and best illustrated by early twin studies [62]. Since then, many TB susceptibility and resistance loci have been reported in humans [63]. Except for a few extreme and very rare cases of Mendelian disorders, TB is a classical example of a complex disease in which each individual TB-associated genetic locus contributes a small proportion of the observed variation in disease susceptibility. Ten years ago, a theoretical study explored whether the natural selection for resistance to TB could have been sufficiently intense to account for the sharp decrease in TB-associated mortality during the later half of the nineteenth century in Europe [3]. The authors concluded that even when applying a time window of 300 years, time would not have been enough to increase the frequency of TB resistance loci to any significant level in these European populations [64]. However, we now know that TB has been affecting humans for millennia, albeit perhaps imposing lower mortality on low-density hunter–gatherer populations (figure 7).

One of the most important questions in TB research is why only approximately 10 per cent of latently infected individuals develop active disease any time during their lifetime [12]. If we knew the answer to this question, we would be in a much better position to design an effective TB vaccine [65]. From an evolutionary point of view, the low attack rate in active TB suggests that MTBC is not a particularly good pathogen in terms of causing disease, yet, considering the 2 billion latently infected individuals, MTBC is extraordinarily successful at generating secondary infections once active TB develops. On a more provocative and highly speculative note, infection with MTBC might in fact be beneficial to at least some human beings (i.e. individuals who never develop active TB), perhaps because a low level of constant immune-stimulation might protect against other diseases. It is well known that the BCG vaccine strain is a potent immune adjuvant which is used to treat bladder cancer. Nevertheless, considering the high global morbidity and mortality owing to TB, it would seem inappropriate to label MTBC as a mere commensal or even a symbiont.

Getting back to the coevolutionary question, some evidence is gradually accumulating which suggests that human genetic variation is indeed interacting with MTBC diversity. At least four recent studies have reported associations between particular human genetic variants in immunity-related genes and different MTBC lineages [66–69]. For example, one study in Vietnam found that a particular SNP in Toll-like receptor 2, a molecule known to be key in innate immune recognition of MTBC, was associated with infection by MTBC lineage 2 in TB patients. Future studies will tell whether these associations reflect reciprocal genetic changes characteristic of host–pathogen evolution [46].

## 9. Concluding remarks

In summary, current evidence suggests that human TB originated in Africa and that MTBC has been associated with its human host for a very long time. It is therefore plausible that this extended host–pathogen association has facilitated some degree of coevolution, and data are accumulating that support this notion. Furthermore, changes in human demography could also have played a role in the evolution of MTBC's life history. The thought that ‘modern’ MTBC has gained an evolutionary advantage compared with ‘ancient’ strains is supported by the larger geographical spread of the former. In addition, ‘ancient’ MTBC lineages appear to be more constrained to their particular geography and associated human populations, while ‘modern’ strains seem to be more promiscuous. Hence from an ecological point of view, ‘ancient’ strains might be referred to as ‘specialists’, while ‘modern’ MTBC might represent ‘generalists’ [70]. Looking into the future and considering the continued global human population increase, coupled with ongoing globalization and urbanization, one wonders where the evolution of human TB is headed. Will MTBC become even more virulent and will particular strain variants become the dominant forms all over the world [71]? Although WHO has devised an ambitious plan to eliminate TB as a global public health problem by 2050 [72], some of the questions discussed here, while primarily of academic interest, should be taken into account by TB researchers and public health officials alike [73]. Answering them will help develop better tools and strategies to control one of the world's oldest and yet still most important human diseases.
